# The Effects of Lubricooling Ecosustainable Techniques on Tool Wear in Carbon Steel Milling

**DOI:** 10.3390/ma16072936

**Published:** 2023-04-06

**Authors:** Nagore Villarrazo, Soraya Caneda, Octavio Pereira, Adrian Rodríguez, Luis Norberto López de Lacalle

**Affiliations:** Aeronautics Advanced Manufacturing Center (CFAA), Faculty of Engineering Bilbao, University of the Basque Country, 48013 Bilbao, Spainnorberto.lzlacalle@ehu.eus (L.N.L.d.L.)

**Keywords:** MQL, CryoMQL, ecofriendly machining, tool wear, carbon steel, carbide tool

## Abstract

This research analyses the viability of using cryogenic cooling combined with MQL (minimum quantity lubrication) lubrication, under CryoMQL technology, as a cutting fluid in the industrial environment to justify the increase in the environmental footprint generated by its use compared to MQL in stand-alone mode. For this analysis, a set of milling tests were carried out on carbon steel AISI 1045, which is one of the most commonly used materials in the business day-to-day. In this set of tests, the evolution of cutting edge wear and energy consumption of both technologies were recorded to check their tool life through technological and environmental analysis. Thus, we sought to discern whether the energy savings derived from the machining process make up for the greater environmental footprint initially generated by the use of CryoMQL technology itself. The results obtained show how the use of CryoMQL not only increased tool life, but also allowed an increase in productivity by increasing cutting speeds by 18%; in other words, thanks to this technology, a more technologically advanced and environmentally friendly process is obtained. By increasing tool life by 30%, a reduction in energy consumption is achieved together with cost savings, which implies that ECO2 machining has economic and ecological benefits.

## 1. Introduction

Faced with the necessity of reducing the use of conventional (flood) cutting fluids, the machining sector’s current aim is the environmental enhancement of production processes by replacing conventional oil emulsion with ecofriendly coolant methods, such as minimum quantity lubrication (MQL) or cryogenic cooling. The cost of cutting fluid can account for up to 17% of manufacturing budgets, surpassing even the cost of cutting tools [1].

Pereira et al. [2] analyzed the environmental impact of different lubrication techniques, focusing on aspects, such as global warming, smog, cutting forces, and machining length, that are strongly associated with life cycle assessment (LCA). One conclusion obtained was that stand-alone techniques were not a viable replacement for conventional lubrication; this study obtained the best technological results by combining some techniques, which are known as CryoMQL.

The principal goal of MQL is minimizing the amount of cutting fluid required to maintain the accuracy and efficiency of manufacturing processes. MQL involves the use of microparticles of oil, which obtain a mist into the cutting area. This technique combines air pressure and a minimal amount of oil, while the air is using for cooling and the oil is used as lubricant as it reduces friction [2]. Through the use of this technology, more sustainable and clean production processes were developed in the industrial environment. He et al. [3] considered MQL to be the most widespread and promising technology for green processing, with economic, health, environmental, feasibility and processing efficacy advantages among others. However, it was also discovered that combining multiple technologies achieves complementary advantages, particularly when using cryogenic technologies.

The main objective of cryogenic cooling is to reduce the temperature in the cutting area, guaranteeing a greater stability in this zone and allowing an increase in the cutting speed during machining; two of the most used fluids in this process are liquid nitrogen (LN_2_) and carbon dioxide liquefied (CO_2_). Cryogenic cooling provides the heat sink required to reduce the tool temperature, while MQL reduces the cutting energy [4]. The best results are obtained when both fluids are injected near the cutting zone. Several studies were analyzed that compared the use of MQL as a stand-alone method and in combination with cryogenic cooling. CryoMQL achieve a balance between ecological and technical factors.

Pereira et al. [5] tested cutting fluids in AISI 304 and obtained a 30% increase in the tool life with CryoMQL; the cryogenic fluid used was CO_2_, while the roughness improved by 40% compared with the theoretical value. Khanna et al. [6] analyzed different cutting fluid strategies in the turning application of 15–5 Precipitated Hardened Stainless Steel; the MQL technology consumed 1% more energy in comparison with liquid CO_2_.

Yıldırım et al. [7] researched these technologies in Ni-based superalloy Inconel 625 to analyze the effects on roughness, cutting temperatures and tool wear. CryoMQL gave the lowest surface roughness and tool wear; CryoMQL reduced cutting temperatures by 24.9% and minimized friction, thus improving the tool life. Kaynak et al. [8] obtained a significant improvement in the surface integrity characteristics and an increase in the tool life.

Past studies of CryoMQL’s use in cutting fluid in milling processes have also have obtained superior performance by using hybrid cooling techniques, which implies that cryogenic cooling and MQL lubrication perform more effectively together compared to standalone tests [9,10]. Lai et al. [11] found that Cryo-LN only produced abrasion, while other technologies also produced adhesion; therefore, they suggested that temperature reduction produces an anti-adhesion capability in 17-4PH stainless steel.

Other studies have also investigated the use of wet, MQL, cryogenic cooling and CryoMQL on these and other difficult-to-machine alloys [12,13,14,15,16,17]; past researchers focused on heat-resistant materials used in the aeronautical sector. These studies mainly concluded that CryoMQL improves productivity by reducing tool wear and cutting forces compared to flood lubrication. Siavaiah & Chakradhar [18] obtained maximum reductions of 53%, 78%, 35% and 16% in tool flank wear, cutting temperatures, surface roughness and cutting force compared to wet machining. The experimental results also showed that cryogenic machining significantly improved machining performance and product quality, even at high feed rates. However, the environmental impacts of lubricooling technologies were not analyzed in industrial common materials, such as carbon steels. Thus, it is important to perform a study with the aim of reducing environmental impact by using CryoMQL techniques in industrial facilities.

This study analyses the viability of using cryogenic cooling combined with MQL lubrication as a cutting fluid in the industrial environment. For this analysis, a set of milling tests were carried out on carbon steel AISI 1045, which are most commonly used industrial materials. In this set of tests, the evolution of cutting edge wear and energy consumption of both technologies was recorded to check tool life through technological and environmental analyses. The results show that CryoMQL decreases tool wear by almost 30% and obtains more stability during milling processes that use a homogenous wear.

## 2. Materials and Methods

A series of tests were carried out on an Ibarmia ZV25 machining center. The material used for the tests was carbon steel AISI 1045 with 220 Brinell hardness, the chemical composition for which is given in Table 1. The tool used was a milling tool with internal coolant and double inserts, which creates a 16 mm diameter cutting tool. The tool body’s total length was 85 mm and the inserts were parallelogram with an angle of 85° and measurements of 12 × 7 × 36 mm. The inserts used were Mitsubishi AOMT1233612PEER-M TiAlCrN-coated sintered carbide inserts of MP6120 grade, with a 20° of incidence angle and 90° of position angle. A researcher analysis of inserts coated in steel H13 obtained the least wear, which was about a 10% [19].

During the tests, the cutting forces were recorded using a triaxial dynamometer table Kistler^®^ 9955A and real-time signal analyzer OROS^®^ OR35 with a sampling rate of 12,800 data/s. Several stops were also made during the tests to record the wear of the insert using a PCE-200 microscope. The final wear was then analyzed in Scanning Electron Microscopy (SEM) Tescan Vega 4 Compact^®^ with the aim of measuring the wear and any possible adhesion.

The cutting conditions used are shown in Table 2. The conditions’ values were determined based on previous experience; we started with industrial cutting conditions and then increased them by 12% and 18%, respectively, with the aim of evaluating the influence of cooling/lubrication technologies under more aggressive cutting conditions. Each test was carried out three times and the average values were used for analysis to eliminate any possible bias due to the random nature of the machining process.

**Figure 1:**
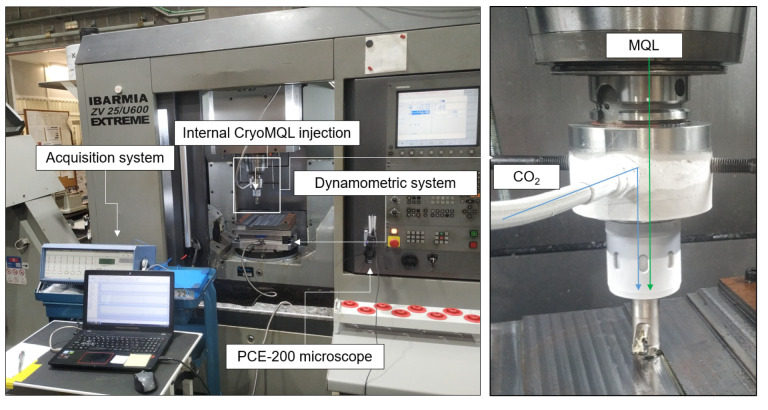
Milling set up.

Regarding the cutting fluids used, MQL and CryoMQL techniques were compared. In the case of MQL technology, a canola biodegradable oil was sprayed with a flow rate of 100 mL/h. In the case of CryoMQL lubricooling technique, MQL was combined with CO_2_ cryogenic using an injection pressure of 10 bar, achieving a biodegradable oil aerosol cryogenized which, once it reached the tool tip, had a cutting temperature of −78 °C. To achieve this effect, a special tool holder was used, which allowed the combining of both fluids without freezing the biodegradable microparticles; it then achieved the cutting tool tip. The test’s setup is shown in Figure 1.

## 3. Results and Discussion

Machinability criteria, such as cutting temperature, surface integrity, surface roughness, etc., are dependent on tool wear; therefore, the research focused on the wear produced along the machining, while the cutting force was analyzed because the two factors are directly related. When faced with an increase in the tool wear, the cutting forces also grew.

Table 3 shows the root mean square of the module of the three cutting forces in the cut length in which the cutting speed was increased. As shown, the difference was minimum; thus, both technologies maintain an almost identical energy consumption, which was one important factor in determining carbon footprint. In particular, the difference between the values was less than 10%, which was considered negligible [20].

(1) $$x_{RMS}=\sqrt{\frac{1}{n}\left(x_{1}^{2}+x_{2}^{2}+\ldots +x_{n}^{2}\right)}$$

(2) $$F_{RMS}=\sqrt{\frac{1}{3}\left(F_{x}^{2}+F_{y}^{2}+F_{z}^{2}\right)}$$

**Figure 2:**
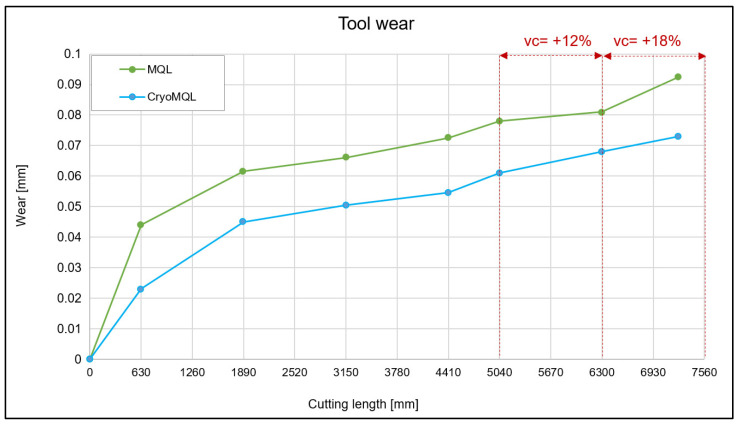
Tool wear due to cutting length.

After the cutting forces analysis, the wear obtained throughout the test was analyzed. During the test, several images of the inserts were taken to analyze the tool. Above all, the wear before and after the changes in the cutting conditions were analyzed. This study stage was the most interesting as, in the face of an increase in productivity, it was also possible to reduce energy consumption. As shown in Figure 2, from the beginning, the wear produced in the MQL test was greater than that produced in the CryoMQL; however, the wear remains similar in both cases and, therefore, the slope is similar, except in the last test stage from the 6300 mm cut length to 7245 mm, where it is appreciated that the MQL had greater wear. At the end of the test, the wear was 27% higher in the case of MQL, whereas in the previous passes the maximum difference was approximately 20%; therefore, CryoMQL had less wear.

**Figure 3:**
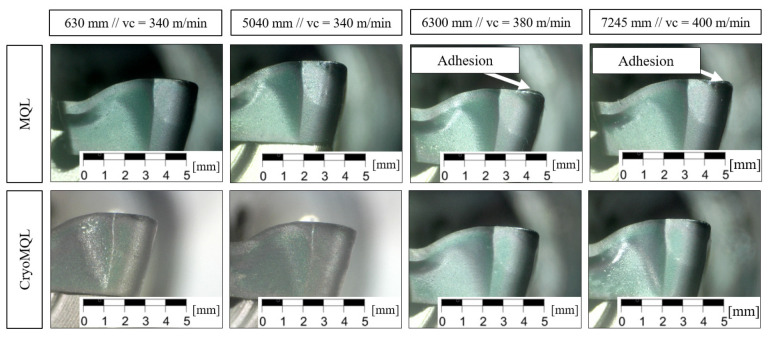
Tool wear.

By analyzing the images taken during the test shown in Figure 3, it is clear that under industrial cutting conditions, homogeneous wear was obtained with both MQL and CryoMQL. However, when the cutting speed increased (cutting length: from 5040 mm to 7245 mm), the wear remained homogeneous using CryoMQL, whereas in the case of MQL, signs of adhesion appear on the edges.

Once tool wear images obtained with the PCE-200 microscope were analyzed, a tool wear analysis was carried out with the SEM microscope to obtain more precise images, verify the materials of which the coating was composed and observe the adhesion in the tool wear area in more detail.

Firstly, in Table 4, the tool coating was analyzed and found to obtain Aluminium, Chrorioum, Nitrogen and Titanium, corresponding with manufacturer specfications.

**Figure 4:**
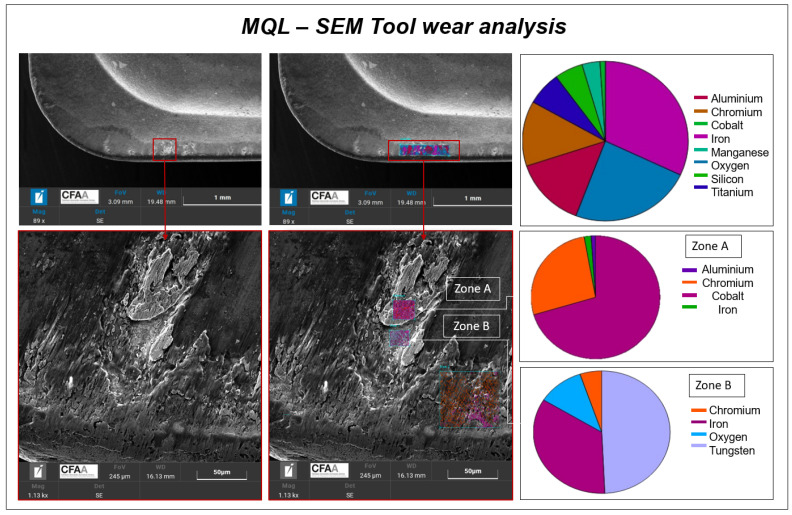
MQL tool wear material analysis.

Figure 4 shows materials in the tool wear. In this figure, it can be appreciated that the wear produced during the MQL test on the cutting edge the coat disappears in some zones that appreciate visually due to the color change, the red square shows a zoom made of the wear area. In these areas, it is shown that the quantity of iron increased and the coat materials decreased. In fact, by zooming into the weariest zone (zone A), it can be observed that the light zones that contain crater-like elements are majority tungsten, which is the main material in carbide tools. In the other zone (zone B) where adhesion is shown, this composition is almost exclusively iron, which means that the machining material AISI 1045 steel is stacked to the tool and, effectively, there is adhesion effect.

**Figure 5:**
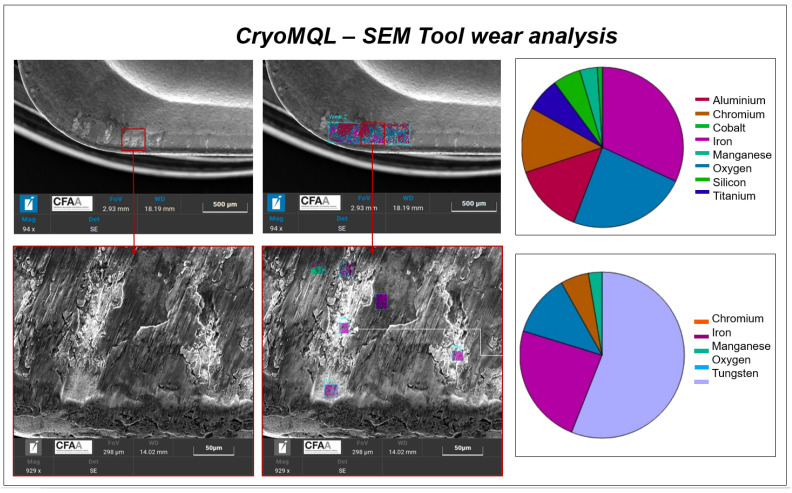
CryoMQL tool wear material analysis.

Finally, we also analyzed the tool wear under CryoMQL conditions. The results obtained are shown in Figure 5. In this case, similar results were obtained because athough the wear was reduced, the effects that cause it were the same. It should be noted that, in this case, the coating composition in some areas decreased due to adhesion wear effects. This phenomenon occurred as iron values increased in those areas. Nevertheless, when comparing the percentage of iron obtained when MQL is applied as cutting fluid or CryoMQL, the results show that the iron present in the tool when CryoMQL was applied was 7% lower than when MQL (zone B) was applied. Thus, the cooling and lubricating properties of CryoMQL implied a decrease in the adhesion phenomenon and, therefore, a reduction in tool wear reduction, as was shown above. Even though the quantity of iron was not directly associated with wear, it was linked to the adhesion phenomenon. This results in decreased stability during cutting and produces an increase in tool wear.

This measurement verifies results obtained through microscopy as it allows for the observation of the wear produced in the insert and the adhesion phenomenon. In the case of MQL, adhesion is greater after machining 6300 mm and starting with more aggressive cutting conditions, such as a cutting speed of 380 mm/min.

Therefore, taking into account the parameters analyzed, it is shown that the use of CryoMQL lubricooling technique balances environmental and technical issues. In particular, although using CryoMQL implies the use of CO_2_ and, thus, an increase in the environmental footprint in comparison with the MQL lubrication technique, its use allows increasing cutting speed and, therefore, reduces manufacturing times without increasing energy consumption. The CryoMQL technique is, thus, a more technologically efficient process, which can be introduced into industrial facilities with aim of advancing towards an ECO2 manufacturing process.

## 4. Conclusions

In this research, CryoMQL technology influence was compared with MQL one in AISI 1045 carbon steel milling. The use of CryoMQL lubricooling technique improved the cutting conditions without increasing mechanical stresses on the tool.

All in all, the following conclusions can be drawn from the tests carried out:

- The cutting forces are similar in both tests, which means that CryoMQL technology does not increase energy consumption;
- The results obtained show how the use of CryoMQL technology implies an increase in productivity through an 18% increase in cutting speed, which implies a reduction in manufacturing times;
- Joining the previous advantage with an increase of 30% in the tool life, which is directly related to cost reductions, can be considered a great improvement for the machining sector. By reducing the use of inserts, it is also possible to reduce their environmental footprint during both the machining and production processes;
- During the tests, adhesion was obtained on the edge of the tool using MQL, which reduced the tool’s stability. On the other hand, using CryoMQL technology, the wear caused in the cutting edge was more homogeneous;
- The adhesion produced during the MQL test was verified with the SEM; when checking the composition in that area, a large increase in iron was observed, whereas in CryoMQL technology a similar increase did not happen. Thus, with the use of CryoMQL technology, adhesion phenomenon was reduced drastically.

Therefore, taking into account the results obtained, the medium-term viability of using cryogenic cooling combined with MQL lubrication as a cutting fluid in the industrial environment can be achieved without affecting current productivity. 

## Figures and Tables

**Table 1:** AISI 1045 material chemical composition.

Chemical Composition %	C	Mn	Si	P	S
AISI 1045	0.40–0.50	0.50–0.80	0.15–0.40	<0.035	<0.035

**Table 2:** Test cutting parameters.

Tests Carried Out			
Length cut stage	0–5040 mm	5040–6300 mm	6300–7245 mm
Cutting speed (vc)	340 m/min (6000 rpm)	380 m/min (6700 rpm)	400 m/min (7000 rpm)
Feed per tooth (f_z_)	0.15 mm/tooth	0.15 mm/tooth	0.15 mm/tooth
Axial depth (a_p_)	3 mm	3 mm	3 mm
Radial depth (a_e_)	9 mm	9 mm	9 mm
Cutting length (L_c_)	315 mm	315 mm	315 mm

**Table 3:** Cutting forces.

RMS Cutting Forces [N]	630 mm vc = 340 m/min	5040 mm vc = 340 m/min	6300 mm vc = 380 m/min	7245 mm vc = 400 m/min
MQL	178,837	188,341	189,532	192,127
CryoMQL	179,325	185,394	192,346	195,338

**Table 4:** Coating chemical composition.

**Base Material**	**Weight %**	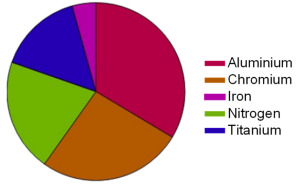
Aluminium	33.5%	
Chromium	26%	
Iron	5%	
Nitrogen	20.5%	
Titanium	15%	

## Data Availability

Not applicable.

## References

[1] Klocke F.A.E.G., Eisenblätter G. (1997). Dry Cutting. CIRP Ann..

[2] Attanasio A., Gelfi M., Giardini C., Remino C.A.R.L.O. (2006). Minimal quantity lubrication in turning: Effect on tool wear. Wear.

[3] Pereira O., Rodríguez A., Fernández-Abia A.I., Barreiro J., de Lacalle L.L. (2016). Cryogenic and minimum quantity lubrication for an eco-efficiency turning of AISI 304. J. Clean. Prod..

[4] He T., Liu N., Xia H., Wu L., Zhang Y., Li D., Chen Y. (2023). Progress and trend of minimum quantity lubrication (MQL): A comprehensive review. J. Clean. Prod..

[5] Damir A., Shi B., Attia M.H. (2019). Flow characteristics of optimized hybrid cryogenic-minimum quantity lubrication cooling in machining of aerospace materials. CIRP Ann..

[6] Khanna N., Shah P., Sarikaya M., Pusavec F. (2022). Energy consumption and ecological analysis of sustainable and conventional cutting fluid strategies in machining 15–5 PHSS. Sustain. Mater. Technol..

[7] Yıldırım Ç.V., Kıvak T., Sarıkaya M., Şirin Ş. (2020). Evaluation of tool wear, surface roughness/topography and chip morphology when machining of Ni-based alloy 625 under MQL, cryogenic cooling and CryoMQL. J. Mater. Res. Technol..

[8] Kaynak Y., Lu T., Jawahir I.S. (2014). Cryogenic Machining-Induced Surface Integrity: A Review and Comparison with Dry, MQL, and Flood-Cooled Machining. Mach. Sci. Technol..

[9] Sivaiah P., Chakradhar D. (2020). Identifying the effectiveness of manner of cryogenic coolant supply in different cryogenic cooling techniques in turning process—A review. Mach. Sci. Technol..

[10] Sivaiah P., Chakradhar D. (2018). Multi performance characteristics optimization in cryogenic turning of 17-4 PH stainless steel using Taguchi coupled grey relational analysis. Adv. Mater. Process. Technol..

[11] Lai Z., Wang C., Zheng L., Lin H., Yuan Y., Yang J., Xiong W. (2020). Effect of cryogenic oils-on-water compared with cryogenic minimum quantity lubrication in finishing turning of 17-4PH stainless steel. Mach. Sci. Technol..

[12] Pereira O., Rodríguez A., Calleja-Ochoa A., Celaya A., de Lacalle L.L., Fernández-Valdivielso A., González H. (2022). Simulation of Cryo-cooling to Improve Super Alloys Cutting Tools. Int. J. Precis. Eng. Manuf.-Green Technol..

[13] Khanna N., Shah P., de Lacalle LN L., Rodríguez A., Pereira O. (2021). In pursuit of sustainable cutting fluid strategy for machining Ti-6Al-4V using life cycle analysis. Sustain. Mater. Technol..

[14] Shokrani A., Al-Samarrai I., Newman S.T. (2019). Hybrid cryogenic MQL for improving tool life in machining of Ti-6Al-4V titanium alloy. J. Manuf. Process..

[15] Rodríguez A., Calleja A., de Lacalle L.L., Pereira O., Rubio-Mateos A., Rodríguez G. (2021). Drilling of CFRP-Ti6Al4V stacks using CO_2_-cryogenic cooling. J. Manuf. Process..

[16] Pereira O., Urbikain G., Rodríguez A., Fernández-Valdivielso A., Calleja A., Ayesta I., de Lacalle L.L. (2017). Internal cryolubrication approach for Inconel 718 milling. Procedia Manuf..

[17] García-Martínez E., Miguel V., Martínez-Martínez A., Manjabacas M.C., Coello J. (2019). Sustainable Lubrication Methods for the Machining of Titanium Alloys: An Overview. Materials.

[18] Sivaiah P., Chakradhar D. (2017). Influence of cryogenic coolant on turning performance characteristics: A comparison with wet machining. Mater. Manuf. Process..

[19] Fox-Rabinovich G.S., Kovalev A.I., Aguirre M.H., Beake B.D., Yamamoto K., Veldhuis S.C., Endrino J.L., Wainstein D.L., Rashkovskiy A.Y. (2009). Design and performance of AlTiN and TiAlCrN PVD coatings for machining of hard to cut materials. Surf. Coat. Technol..

[20] Tabernero I., Lamikiz A., Martínez S., Ukar E., De Lacalle L.L. (2012). Modelling of energy attenuation due to powder flow-laser beam interaction during laser cladding process. J. Mater. Process. Technol..

